# Supplementary material for: Large Gender Gap in Oral Hygiene Behavior and Its Impact on Gingival Health in Late Adolescence
Source: Int J Environ Res Public Health. 2020 Jun 18;17(12):4394. doi: 10.3390/ijerph17124394 (PMC7345042; doi:10.3390/ijerph17124394)
Supplement: Supplementary file 1 [file ijerph-17-04394-s001.pdf]

Frequency of brushing

How many times do you brush a day? Please choose one of the following options.

☐	0
☐	1 time
☐	2 times
☐	3 times
☐	4 times or more

Duration of brushing

How long do you brush each time? Please choose one from the following options.

☐	less than 1 min
☐	1min
☐	2 – 3 min
☐	4 – 5 min
☐	6 min or more
